# Pilot validation of a verbal practical judgement assessment (VPJ) among community-dwelling older adults in Israel: the first step toward a national standard

**DOI:** 10.1590/1980-5764-DN-2022-0047

**Published:** 2023-05-29

**Authors:** Yael Zilbershlag

**Affiliations:** 1 Ono Academic College, Faculty of Health Allied Professions, Department of Occupational Therapy, Kiryat Ono, Israel.

**Keywords:** Safety, Cognition, Executive Function, Frail Elderly, Outcome Assessment, Health Care, Segurança, Cognição, Função Executiva, Idoso Fragilizado, Avaliação de Resultados em Cuidados de Saúde

## Abstract

Increased longevity and subsequent increase in older populations emphasize the importance of assisting older people to continue living in safe and residential situations for as long as possible. Judgement, an important aspect of cognition, and a predictor of function may become impaired and compromise safe living. Yet, judgement is difficult to assess, and few valid instruments are utilized in clinical settings that accurately evaluate judgement in older people.

**Objectives:**

This pilot study aimed to translate, culturally adapt, and initiate the validation of the Hebrew version of the verbal practical judgement (VPJ) assessment among community-dwelling older people.

**Methods:**

A total of 50 older adults, aged over 65 years, living in the community in Israel, half of whom were independent (n=27, 54%), and the rest dependent participants in a day centre with some level of cognitive/functional decline, completed the VPJ evaluation and comparison assessments.

**Results:**

Positive and significant (p<0.05) relationships between VPJ and standard assessments were found, demonstrating convergent validity. By comparing VPJ scores between independent and dependent older adults, results also supported discriminant validity. Finally, a multiple hierarchical regression demonstrated a positive relationship between instrumental activities of daily living and judgement.

**Conclusions:**

This pilot study found the VPJ feasible, likely valid, and culturally adaptable to assess judgement in Israeli older adults. Assessing judgement will provide older adults and their families with essential information regarding function, cognition, and safety and will enable them to live/return home in accordance with their autonomy, safety, and well-being.

## RESUMO.

O aumento da longevidade e o subsequente aumento das populações mais velhas enfatizam a importância de ajudar os idosos a continuar vivendo em situações residenciais seguras pelo maior tempo possível. O julgamento, um aspecto importante da cognição e um preditor da função, pode ser prejudicado e comprometer uma vida segura. No entanto, o julgamento é difícil de avaliar e poucos instrumentos válidos são utilizados em ambientes clínicos que avaliam com precisão o julgamento em pessoas idosas.

**Objetivos:**

Este estudo piloto teve como objetivo traduzir, adaptar culturalmente e iniciar a validação da versão hebraica da avaliação *Verbal Practical Judgment* (VPJ) entre idosos residentes na comunidade.

**Métodos:**

Cinquenta idosos, com mais de 65 anos, residentes na comunidade em Israel, metade dos quais eram independentes (n=27, 54%) e o restante participantes dependentes de um centro diurno com algum nível de declínio cognitivo/funcional, completaram o VPJ avaliações de avaliação e comparação.

**Resultados:**

Foram encontradas relações positivas e significativas (p<0,05) entre VPJ e avaliações padrão, demonstrando validade convergente. Ao comparar os escores VPJ entre idosos independentes e dependentes, os resultados também apoiaram a validade discriminante. Finalmente, uma regressão hierárquica múltipla demonstrou uma relação positiva entre AIVD e julgamento.

**Conclusão:**

Este estudo piloto considerou o VPJ viável, provavelmente válido e culturalmente adaptável para avaliar o julgamento em idosos israelenses. A avaliação do julgamento fornecerá aos idosos e suas famílias informações essenciais sobre função, cognição e segurança e os capacitará a viver/voltar para casa de acordo com sua autonomia, segurança e bem-estar.

## INTRODUCTION

In both developed and developing countries, people are living longer than they were even a decade ago^1^. There is a normal rate of related cognitive decline that occurs with age^2^; this rate of decline may be exacerbated by the increase in neurocognitive disorders that also develop as we age^3^. These two occurrences predict a large global population well over the age of 60 years with both normal and pathological rates of cognitive impairment^1^. Research indicates that cognitive decline is significantly associated with limitations in basic activities of daily living (BADL) and instrumental activities of daily living (IADL), especially executive functioning^4,5^. The challenges of declining cognition and impaired functional performance among older adults in general, and individuals living at home (community dwelling) in particular raise concerns about safety, quality of life, and dependence on health care services^6,7^.

Executive functions are meta-cognitive processes that enable goal-directed behaviours, including flexibility of mental processes, inhibition, fluency, working memory, strategy, abstract thinking, problem-solving, and judgement, all of which enable the performance of complex tasks of daily life^8,9^. Judgement is one of the essential components of executive function, yet it is not frequently assessed, perhaps due to its complexity as a cognitive skill^10^. Judgement is defined as the ability to assess a situation and make appropriate decisions, based on relevant information, context, possible alternative solutions, and understanding of the outcomes. Deficits in the ability to judge can compromise a person's safety and function and could indicate an increased need for external support^11^. It has been found that both decreases in cognitive and functional status are associated with decreased ability in judgement^12^. Therefore, assessment of the client's judgement ability may help the provider identify and track preclinical stages of dementia, as well as help protect the client's financial or medical harmful events^13^. Assessing judgement can help the health care provider ascertain the older client's future capability of independent living at home^14^.

Yet recent research has demonstrated that many practitioners do not feel confident in their ability to assess judgement^15,16^. This deficiency necessitates a cadre of validated and reliable assessment tools. Yet, there are few judgement assessment tools developed for the older adult population^12,16,17^. The main, validated assessments of judgement in use appear to be the Cognistat^18^, Kitchen Picture Test (KPT)^19^, The Judgment Assessment Tool (JAT)^20^, Test of Practical Judgment (TOP-J)^21^, and the Verbal Test of Practical Judgment (VPJ)^16^. Only the TOP-J and the VPJ focus on real-world scenarios that can be applied easily in a clinical setting. The VPJ also uses less complex sentence structures and was thought to be more suitable for translation and validation. The VPJ enables the provider to effectively evaluate the patient's ability to judge in a clinical setting and measure the associated effect of judgement with function and safety. In this way, providers can assess practical judgement in older adults, which provides vital information about judgement status and helps determine appropriate intervention, while maintaining and optimizing safety. As this instrument was validated on an English-speaking population, it was necessary to apply it to Hebrew-speaking population in Israel, due to differences in language and culture^22^.

The objective of this pilot study was to translate, culturally adapt, and initiate a validation process of a Hebrew version of the VPJ among community-dwelling older Israeli people.

## METHODS

### Participants

For this pilot study, two distinct groups were selected using a convenience sample; one group lived independently in the community (n=27), and dependent older adults were participants in a day centre (n=23). In Israel, older people in need of support in their BADLs or IADLs are eligible to participate in a day centre to offer them additional support^23^. Therefore, the day centre participants would have more cognitive decline as compared to independent participants. These two groups were selected in order to widen the scope of cognitive ability and subsequent validation process. Independent participants were mobile both in and out of the home and capable of independent BADLs and IADLs. For them, study eligibility was determined by observation and a brief interview, and data were collected in their homes. The participants from the day centre were also living in the community but had some level of measurable cognitive/functional decline. They were surveyed in the day centre. Ethical approval was received from the participating academic institution (#202008ono); participants received an explanation of the study and signed informed consent. In order to include a wider range of participants cognitive status, we used the Montreal Cognitive Assessment (MoCA)^24^ with a cutoff score of >14, which excluded only those participants with more severe cognitive decline^25^. There were no other exclusion criteria.

Participants were recruited by occupational therapy student researchers, who either approached possible participants during activities at the day centre or at participants’ homes in a nearby community. The student researchers were trained by the primary investigator to administer the evaluations and conducted all participant interviews.

### Procedure

Cross-cultural validation was conducted using the accepted standards of translation, back translation, expert review, and pretesting^22^.

### Instruments

#### Study variable

The VPJ^16^ assesses a person's practical judgement and is intended for use among older adults:

- It consists of 10 questions requesting participants’ reactions to certain scenarios.
- Participants are scored for correct answers. Each question receives points between 0 (incorrect) and 2 (correct), with 1 point awarded for a partial answer. For example, for the question, “Suppose you realize that you accidentally took too much of your medication. You took twice the prescribed dose. What would you do?,” correct answer “I would call my medical provider”=2 points; partial answer “I would wait and see if I were sick and needed help”=1 point, and “I would do nothing” or similar=0 points.
- Scores set by Mansbach et al.^16^ are as follows: the range 0–12 indicates severe impairment in practical judgement; 13–15 indicates some impairment; and 16–20 indicates no apparent impairment. The original assessment had high inter-rater reliability (r=0.99).

#### Comparison variables

The comparison variables were chosen in accordance with the original validation by Mansbach et al.^16^

#### Basic and instrumental functioning (basic activities of daily living and instrumental activities of daily living)

#### Basic activities of daily living questionnaire (Barthel Index)^26^

This questionnaire evaluates the assessment of basic functioning of daily life. The final score ranges from 0 to 100. Higher score denotes better functioning. Internal consistency found in this study: Cronbach's α=0.82.

#### Instrumental activities of daily living scale (Lawton IADL)^27^

Functional tools aimed to assess functioning in everyday instrumental areas. Complete score ranges from 0 to 23; the higher the score, the better the functional condition. Internal consistency in this study: Cronbach's α=0.87.

#### Cognitive functioning

#### Performance assessment of self-care skills (PASS-Home)^28^

It is a performance-based observational measure that assesses daily functioning essential for community living. The PASS consists of 26 tasks in 4 domains: functional mobility, personal self-care, cognitive (CIADL), and physical (IADL) tasks. In our study, two CIADL tasks were included: telephone use and medication management. Score ranges from 0 to 3, with 3 indicating independent.

#### Cognition

#### Montreal cognitive assessment^24^

MoCA examines cognitive abilities. The maximum score is 30 points.

#### Executive functioning

#### Executive clock drawing test (CLOX)^29^

This is a screening test for executive functioning. The maximum score is 15 points; lower score indicates greater impairment. A cutoff point for CLOX1 and CLOX2 is ≥9 and ≥12, respectively.

#### Depression

#### Geriatric depression scale (GDS15)^30^

This is a self-report depression screening tool for older adults, with score ranging from 0 to 15. A score of ≥11 is considered a cutoff value for extreme depression. Good internal consistency in this study: Cronbach's α=0.80.

### Data analysis

Data were analysed using SPSS (version 28; IBM, Chicago, IL, USA). Background characteristics were described with frequencies and percentages, or means and standard deviations, according to variable distribution. Kendall's W was calculated to assess inter-rater agreement for the VPJ items, and Cronbach's α was used for internal consistencies. Total scores were computed using summaries or means of the items per instrument. Item-level statistics for the VPJ were described using means, standard deviations, skewness and kurtosis, inter-item correlations, and Cronbach's α if the item was deleted. Spearman's correlation test and t-test were used to calculate the relationships between the VPJ and background characteristics. Convergent validity was first examined with Spearman's correlations between the cognitive and functional measures and VPJ. It was then examined with partial correlations, controlling for years of education, and using exponential transformations for negatively skewed variables. Discriminant validity was first examined with a t-test of the VPJ score by independent/decline status. It was then examined with an analysis of variance, controlling for years of education. Second, discriminant validity was examined with a Spearman's correlation between the GDS and VPJ scores, and then with a partial correlation, controlling for years of education and using the logarithmic transformation of the positively skewed GDS variable. Finally, a multiple hierarchical regression was calculated to assess the relationship between IADL functioning and VPJ. Years of education were entered in the first step and VPJ in the second. A significance level was set at 0.05.

## RESULTS

### Steps toward validation of translated and culturally modified VPJ assessment

Approval was obtained from the author^16^ to translate, culturally modify, and initiate the validation of the assessment for a Hebrew-speaking older population in Israel. As is accepted in cross-cultural validation of an instrument^22^, the following steps were completed. The VPJ was translated into Hebrew and back-translated into English. Subsequently, the revised assessment was tested with volunteers in the community. In addition, the assessment was presented to a group of occupational therapists working in a geriatric hospital who provided feedback regarding the wording and cultural applicability of the questions. An open discussion was then conducted among the occupational therapy students, the occupational therapists working in the rehabilitation ward, and the primary investigator, bringing up possible linguistic and cultural relevance issues that had arisen during their piloting of the translated assessment. Six questions were thus revised to reflect these issues (Table 1). These modifications were performed in coordination with the author of the VPJ and additions/changes did not alter the essence of the questions.

**Table 1:** Modifications of original verbal practical judgement questions for this target population.

Question #	Original question	Modification
1	“Suppose you have been taking a medication for a long time?”	The word “essential/crucial/critical/vital” was added before the word medication.
4	“Suppose you plan to microwave a frozen dinner for your meal. When taking the dinner out of the freezer, you notice it is not frozen. What do you do about eating?”	Since in Israel it is less common to eat readymade frozen meals, the question was changed to: “Suppose you plan to cook and eat a meat dish for lunch” [AND?]
6	“Suppose you are waiting for a taxi to take you to an appointment with your doctor. The taxi is 15 minutes late. What should you do?”	Since the health care system works in the country differently from the US, the adjustment was made to refer to a visit to a private physician (who you have been waiting to see for several months) instead.
8	“Suppose you receive a check the beginning of every month that you use to meet your expenses. You have _five days left in the month. The electric bill is due and you have a prescription to pick up. You can't afford to pay both. What should you do?”	The question was adjusted to reflect the public pension that people aged 65+ receive in Israel from the National Insurance Institute/ “Suppose you received your stipend from the National Insurance Institute. The electric bill is due and you have a medication you must buy. You can't afford to pay both. What should you do?”
9	“Suppose you buy a meal at a restaurant that costs $15. You hand the server $20, and she gives you $2 back. What should you do?”	The amounts were adjusted to reflect restaurant meal prices in Israel.
10	“Suppose someone you do not know comes to your door to sell you a magazine subscription. He asks you if he can come into your home to tell you about great magazine discounts. What should you do?’	Since in Israel it is not acceptable for subscriptions to be sold door to door, the question was modified to: “Suppose someone you do not know comes to the door of your house and tells you that he has come to repair/test the gas. You are not aware that there is a problem with the gas. What should you do?”

### Sociodemographic and clinical characteristics

A total of 50 older adults participated in this study (M=78.2 years, SD=9.79, 65–99) (Table 2).

**Table 2:** Socioeconomic and clinical characteristics of the participants (N=50).

		Total sample	Day centre (n=23)	Independent living (n=27)	Significance
Age, M (SD), range		78.2 (9.79), 65–99	85.5 (7.20)	72.0 (7.06)	t(48)=-6.67, p<0.001
Gender, n (%)					
	Male	18 (36.0)	6 (26.1)	12 (44.4)	Z=1.35 p=0.178
	Female	32 (64.0)	17 (73.9)	15 (55.6)	
Marital status, n (%)					
	Married	23 (46.0)	7 (30.4)	16 (59.3)	Z=2.04 p=0.042 (married vs. not married)
	Divorced	7 (14.0)	1 (4.3)	6 (22.2)	
	Widowed	20 (40.0)	15 (65.2)	5 (18.5)	
Place of birth, n (%)					
	Israel	24 (48.0)	9 (39.1)	15 (55.6)	χ^2^(2)=3.69 p=0.158
	Europe America	15 (30.0)	10 (43.5)	5 (18.5)	
	Asia Africa	11 (22.0)	4 (17.4)	7 (25.9)	
	Years of education, M (SD), range	13.2 (3.51), 4–20	11.3 (3.04)	14.8 (3.10)	t(48)=4.00, p<0.001
Level of education, n (%)					
	High school or less	24 (48.0)	16 (69.6)	8 (29.6)	Z=2.82 p=0.005
	Above high school or academic	26 (52.0)	7 (30.4)	19 (70.4)	
Self-rated economic status, n (%)					
	Good/very good	38 (76.0)	14 (60.9)	24 (88.9)	Z=2.31 p=0.021
	Moderate/not good	12 (24.0)	9 (39.1)	3 (11.1)	
Place of residence, n (%)					
	Home	47 (94.0)	20 (87.0)	27 (100.0)	--
	Assisted living facility	3 (6.0)	3 (13.0)	--	
Living with: n (%)					
	Partner	17 (34.0)	6 (26.1)	11 (40.8)	--
	Family	8 (16.0)	1 (4.3)	7 (25.9)	
	Alone	16 (32.0)	7 (30.5)	9 (33.3)	
	Caregiver	9 (18.0)	9 (39.1)	--	
Chronic illness, n (%)					
	Yes	20 (40.0)	12 (52.2)	8 (29.6)	Z=1.62 p=0.105
Medications, n (%)					
	Yes	41 (82.0)	23 (100.0)	18 (66.7)	--
Mobility in the home, n (%)					
	Independent	37 (74.0)	10 (43.5)	27 (100.0)	--
	With aid	13 (26.0)	13 (56.5)	--	
Mobility outside the home, n (%)					
	Independent	30 (60.0)	6 (26.1)	24 (88.9)	--
	With aid	20 (40.0)	17 (73.9)	3 (11.1)	
BADL, M (SD), range		92.7 (13.45), 40–100	85.2 (16.62)	99.1 (3.93)	t(24.10)= 3.91, p<0.001
MoCA, M (SD), range		21.9 (3.94), 14–29	20.1 (3.65)	23.4 (3.54)	t(48)=3.29, p=0.002
CLOX1, M (SD), range		11.2 (3.75), 0–15	9.1 (4.35)	13.0 (1.83)	t(28.58)=3.99, p<0.001
CLOX2, M (SD), range		12.9 (2.39), 6–15	11.8 (2.69)	13.8 (1.66)	t(48)=2.24, p=0.030
IADL, M (SD), range		17.4 (6.30), 3–23	12.1 (5.40)	21.9 (2.04)	t(27.35)=8.22, p<0.001
IADL – medication management (PASS)					
	Independence, M (SD), range	2.4 (0.75), 0–3	2.1 (0.96)	2.7 (0.36)	t(48)=2.73, p=0.009
	Safety, M (SD), range	2.8 (0.51), 0–3	2.7 (0.71)	3.0 (0)	t(22.00)=2.44, p=0.023
	Appropriateness, M (SD), range	2.3 (0.75), 0–3	2.1 (0.81)	2.5 (0.64)	t(48)=1.68, p=0.100
IADL – telephone use (PASS)					
	Independence, M (SD), range	2.6 (0.79), 0–3	2.2 (1.06)	2.9 (0.22)	t(28.02)=3.51, p=0.002
	Appropriateness, M (SD), range	2.5 (0.79), 0–3	2.1 (0.94)	2.8 (0.51)	t(35.90)=2.88, p=0.007
	GDS, M (SD), range	3.1 (3.03), 0–13	3.9 (2.77)	2.4 (3.13)	t(48)=−2.63, p=0.011

Abbreviations: BADL: basic activities of daily living; MoCA: Montreal Cognitive Assessment; CLOX1: Executive Clock Drawing Test Part 1; CLOX2: Executive Clock Drawing Test Part 2; IADL: instrumental activities of daily living; PASS: performance assessment of self-care skills; GDS: Geriatric Depression Scale.

All independent participants were independently mobile at home. About a half of those from the day centre needed some type of mobility assistance at home (56%) and/or assistance outside the home (74%).

BADL mean score for the sample was high, M=92.7 (range 40–100, SD=13.45, median=100), with day centre participants showing somewhat lower scores than the independent participants. Significant differences in favour of the independent participants were found for MoCA, CLOX, IADL, and GDS as well (Table 2).

### Verbal practical judgement description

Inter-rater agreement among 7 participants and 12 judges was high, ranging between Kendall's W=0.71 and Kendall's W=1.0 (the result for each of the 10 items is: Q1–W=0.92, Q2–W=0.95, Q3–W=1.00, Q4–W=0.81, Q5–W=0.80, Q6–W=1.00, Q7–W=0.71, Q8–W=0.76, Q9–W=0.95, and Q10–W=0.92).

Table 3 presents the description and distribution of the items of the VPJ. Results show that three questions, namely, Q3 “Falling in the bathroom,” Q7 “Personal hygiene,” and Q10 “Strangers in the home,” had the highest mean scores related to judgement (M=1.76–1.90). Questions Q6 “Late for appointment” and Q9 “Calculating change” (M=1.42–1.44) had somewhat lower scores. The moderate mean scores were found for Q1 “Medication use,” Q2 “Accidental overdose,” Q4 “Meal preparation,” and Q8 “Balancing the check book” (M=1.06–1.26), and the lowest mean score was found for Q5 “Time management” (M=0.68). Internal consistency was low, with Cronbach's α=0.52. It is similar to the internal consistency found by Mansbach et al.^16^ in their first study with 51 participants (α=0.53) and lower than the one found in their second study with 110 participants (α=0.68).

**Table 3:** Question-level statistics for the verbal practical judgement (n=50).

Items	M	SD	Skewness	Kurtosis	α	Inter-item	Item-total
1. Medication management	1.18	0.63	−0.15	−0.47	0.53	0.05	0.09
2. Accidental overdose	1.06	0.96	−0.12	−1.95	0.53	0.07	0.14
3. Falling in the bathroom	1.80	0.61	−2.75	5.79	0.53	0.06	0.10
4. Meal preparation	1.26	0.83	−0.53	−1.34	0.46	0.13	0.32
5. Time management	0.68	0.96	0.70	−1.58	0.42	0.17	0.41
6. Late for appointment	1.44	0.67	−0.81	−0.43	0.44	0.17	0.43
7. Personal hygiene	1.76	0.43	−1.26	−0.44	0.51	0.06	0.21
8. Balancing the check book	1.18	0.77	−0.33	−1.24	0.50	0.09	0.23
9. Calculating change	1.42	0.70	−0.81	−0.54	0.50	0.10	0.21
10. Strangers in the home	1.90	0.36	−3.96	16.48	0.52	0.04	0.09

Notes: α: Cronbach's α if the item was deleted; inter-item: average inter-item correlation; item-total: corrected correlation with total Verbal Test of Practical Judgment (VPJ) total score. Skewness SE=0.34; Kurtosis SE=0.66.

The total scale score was M=13.68 (range 8–20, SD=3.12, median=13), with a rather normal distribution (skewness=0.12, SE=0.34 and kurtosis=−0.73, SE=0.66). One-third of the participants (n=18, 36%) were classified as having a severe impairment in practical judgement (scores of 8–12), another one-third (n=17, 34%) had some impairment (scores of 13–15), and one-third (n=15, 30%) had no apparent impairment (scores of 16–20).

The total VPJ total score was negatively associated with age (r=-0.32, p=0.024) and positively associated with years of education (r=0.53, p<0.001). The score was unrelated to economic status and did not differ by gender. Age was negatively related to years of education (r=-0.57, p<0.001), meaning the older the participant, the fewer the years of education.

### Convergent validity

Convergent validity was examined with Spearman's correlations between the cognitive and functional measures and VPJ (Table 4). All correlations with functional and cognitive measures were positive and significant (p<0.05), with moderate-to-high coefficients for MoCA, CLOX1, CLOX2, and IADL — medication management (PASS) and low-to-moderate coefficients for IADL — telephone use (PASS).

**Table 4:** Spearman correlations between verbal practical judgement and cognitive and functional measures (n=50).

	MoCA	CLOX1	CLOX2	IADL	IADL – medication management (PASS)			IADL – telephone use (PASS)	
					Independence	Safety	Appropriateness	Independence	Appropriateness
VPJ (r_s_)	0.57	0.56	0.45	0.50	0.52	0.28	0.29	0.35	0.28
p	<0.001	<0.001	0.001	<0.001	< 0.001	0.046	0.044	0.013	0.049

Abbreviations: VPJ: verbal practical judgement; MoCA: Montreal Cognitive Assessment; CLOX1: Executive Clock Drawing Test Part 1; CLOX2: Executive Clock Drawing Test Part 2; IADL: instrumental activities of daily living; PASS: performance assessment of self-care skills.

Using exponential transformations for non-normally distributed variables (negatively skewed) and controlling for years of education, associations with the IADL — measures of medication management (PASS), safety and appropriateness, and telephone use task became nonsignificant (p=0.178 to p =0.678). All positive associations with MoCA, CLOX1, CLOX2, IADL (Lawton), and IADL — medication management (PASS) retained their significance (p=0.047 to p<0.001). These results support the convergent validity of the VPJ in our pilot sample.

### Discriminant validity

Discriminant validity was examined using the VPJ score for the independent (n=27) versus day centre (n=23) participants and the depression (GDS) score. A t-test for the VPJ score, comparing the independent participants with the day centre participants, was found to be significant, with participants defined as independent (M=15.04, SD=3.03), having a higher mean score than participants from the day centre (M=12.09, SD=2.43) (t(48)=3.75, p<0.001, d=1.08). The significance of the difference was retained when controlling for years of education, F(1, 47)=4.30, p=0.044, η^2^=0.084. A Spearman's correlation between the total depression score (GDS) and the VPJ score was not significant (p=0.227), as was the partial correlation with the log-transformed non-normally distributed GDS variables (positively skewed), controlling for years of education (p=0.739).

### Judgement and instrumental activities of daily living

Finally, a multiple hierarchical regression was calculated to assess the relationship between IADL-Lawton functioning and judgement (VPJ) (Table 5). Years of education were significant in the first step, explaining 16% of the variance in IADL functioning. VPJ in the second step was also significant and added 8.5% to the explained variance in IADL functioning, beyond the contribution of years of education.

**Table 5:** A hierarchical regression for instrumental activities of daily living functioning (n=50).

		B	SE	β	p	Adj. R^2^
Step 1						
	Years of education	0.75	0.23	0.42	0.002	0.160
Step 2						
	Years of education	0.41	0.26	0.23	0.128	0.245
	Verbal practical judgement	0.74	0.29	0.37	0.015	

Notes: F(2,47) = 8.93; p<0.001.

## DISCUSSION

Many of our results mirrored the results of the original study^16^, where judgement was not correlated with depression, gender, or marital status, but was correlated with IADL and cognition. Conversely, we did find a correlation between age and judgement and between years of education and judgement. It should be noted that Mansbach et al.^10^ reported a similar correlation with age in their second study (r=-0.41, p<0.001), but not a significant association with the level of education in either of their studies. Our results are consistent with the literature; when there is an increase in age, there is a decrease in executive functions and, among them, judgement.^16,31^

### Judgement, cognition, and functioning

We found a correlation between function, cognition, and judgement, which strengthens our conclusion regarding probable convergent validity. Accordingly, in the study of Hinrich and colleagues^32^, on the relationship between judgement and functional status and ability to perform independent daily activities in a population with dementia, there is a significant relationship between the level of cognition and the functional state, as well as between the functional state of the person and judgement ability. The researchers found that functional status and cognition were jointly associated with 56% of the variance in judgement. That is, as the cognitive ability diminished, the ability to judge and solve problems diminished as well. Moreover, the results of our study indicate that there is a relationship between a person's judgement and the level of function required to achieve independence.

A similar judgement assessment to the one used in this study, with a much larger sample, found that reduction in overall judgement ability was associated with cognitive impairment.^33^ Assessing a person's judgement abilities provides information about the ability to formulate plans and execute them during complex tasks. Patients with executive disability may appear to have difficulty judging and, therefore, will need tailored help to ensure safety and health.

### Discriminant validity

The assessment distinguished between the two groups of participants, independent as compared with dependent. This further strengthens our supposition that judgement/executive functioning is associated with the level of functioning^4,34^.

### Limitations

We used a small convenience sample from one community in one geographic location. To continue validation, a larger and more diverse population is currently being studied. Additionally, we tried to replicate the original validation of the tool, yet some of the instruments used were not available in Hebrew or in Israel. Overall, our modified assessment did have similar, albeit low, internal consistency as the original study. Given our small sample, this is not surprising and is consistent with other judgement assessments. Overall, as this was a brief assessment with multiple areas of inquiry, a high internal consistency was not expected^28^.

In conclusion, this pilot study initiated a validation process of a judgement assessment prior to conducting a nationwide study within various community and hospital settings. Due to the importance of assessing judgement and the lack of a standard accessible instrument^17^, it is crucial that an easily applied assessment must be validated for diverse older populations, with the eventual goal of incorporating it within a standard evaluation. In this pilot study, the VPJ was found feasible, culturally adaptive, and likely valid to assess practical judgement in Israeli older adults in the community. Older adults and their families could then receive essential information regarding function, cognition, and safety and enable them to live/return home in accordance with their autonomy, safety, and well-being.
